# Expression of p120 – catenin in oral squamous cell carcinoma and apparently normal mucosa adjacent to oral squamous cell carcinoma

**DOI:** 10.4317/jced.61057

**Published:** 2024-04-01

**Authors:** Aishwarya Rajeev, Saikumar Katukuri, Shravya Devarashetty, Satyanarayana Dantala, Aishwarya-Lakshmi Billa

**Affiliations:** 1 Associate Professor, Department of Oral Pathology and Microbiology, Mahatma Gandhi Dental College & Hospital, Rajasthan, India; 2 Assistant Professor, Department of Public Health Dentistry, Panineeya Institute of Dental Sciences and Research Centre, Hyderabad, India; 3 Assistant Professor, Department of Public Health Dentistry, MNR Dental College and Hospital, Hyderabad, Sangareddy, India; 4 Associate Professor, Department of Public Health Dentistry, MNR Dental College and Hospital, Hyderabad, Sangareddy, India; 5 Senior Resident, Department of Public Health Dentistry, Government Dental College & Hospital, Hyderabad, India

## Abstract

**Background:**

An essential molecular occurrence in carcinogenesis that can lead to invasion and migration, predisposing cells to malignant transformation, involves alterations in cell adhesion molecules, such as p120 catenin. The destabilization of E-cadherin, caused by the loss or phosphorylation of p120 catenin (p120), regulates cadherin stability and turnover, impacting cell adhesiveness and migratory capacity. Consequently, p120 is associated with the invasiveness and progression of various human epithelial tumor types, including Oral Squamous Cell Carcinomas (OSCC). The present study aimed to assess and establish a correlation between the expression of p120 antibody in OSCC and Apparently Normal Mucosa Adjacent to OSCC (ANMAOSCC).

**Material and Methods:**

The immunoexpression of p120 in 300 selected cases was categorized into two groups: OSCC (n = 150) and ANMAOSCC (n = 150). Two 4µm-thick tissue sections from the selected blocks were prepared. One section was stained with Hematoxylin and Eosin, while the other underwent immunohistochemical (IHC) staining using anti-p120 catenin antibody (clone No. EP66; Catalog No. PR062; PathnSitu, Wayne, PA, USA). The analysis of p120 immunoexpression included parameters such as intensity, percentage, and the location of staining.

**Results:**

In OSCC, over 80% of cases expressed p120, with only 16% exhibiting loss of expression. In ANMAOSCC, all cells maintained p120 expression. In OSCC, p120 was predominantly localized to the membrane and cytoplasm in 76%, while in ANMAOSCC, over 90% showed membrane localization. Regarding positivity, only 19% of OSCC cases reported positivity in >50% of cells, compared to 64.7% in ANMAOSCC. The extent of staining in ANMAOSCC was observed up to the granular layer (45%) and corneal layer (19%).

**Conclusions:**

The atypical staining pattern of p120 may indicate a loss of adhesion and could serve as a marker for identifying the malignant potential of ANMAOSCC and the aggressiveness of OSCC.

** Key words:**Oral Squamous Cell Carcinoma, P120 Catenin, Cell Adhesion Molecules, Gene Expression Regulation.

## Introduction

Oral Squamous Cell Carcinoma (OSCC) is globally recognized as the eighth most prevalent cancer and holds the third position in the south-central Asian region (1-3). More than 90% of cancers arising from the oral mucosa are attributed to OSCC (3). The cancer burden is steadily increasing, particularly in middle and low-income countries (4), with an estimated 405,000 new cases of oral cancer reported annually worldwide (5). Among South East Asian nations, India, Pakistan, and Taiwan record the highest number of cancer cases (6).

Oral Squamous Cell Carcinoma (OSCC) has a multifactorial etiology, with predisposing factors encompassing lifestyle-related components such as the use of tobacco, alcohol consumption, unhealthy dietary habits, and a genetic predisposition. Additionally, factors like trauma, prolonged tissue irritation, radiation exposure, and infections/immunocompetence may also contribute to its multifactorial nature (7). Potentially malignant lesions, such as oral leukoplakia and lichen planus, carry an elevated risk of progressing to OSCC, with epithelial dysplasia serving as a crucial indicator for assessing the risk of malignant transformation (8).

Despite limited progress in prevention and treatment modalities and unchanged survival rates, early diagnosis of OSCC is vital for effective intervention (9,10). There is a growing interest in understanding the molecular and biological events that drive the progression of the epithelium surrounding carcinoma to Squamous Cell Carcinoma (SCC). The concept of field cancerization, introduced by Slaughter in 1953, underscores the importance of examining the epithelium surrounding OSCC for risk assessment and cancer management (11).

One of the unique characteristics of oral epithelial cells is the presence of extensive intercellular junctional complexes and adhesions. Alterations in the level of cell-cell adhesion are the important molecular event in the development of carcinogenesis, and also could result in invasion and migration to distant sites to form new tumors. E-cadherins is a 120-KDa transmembrane glycoprotein responsible for the maintenance of tissue architecture through proper cell-cell adhesion and binding of the intracytoplasmic domains with catenins. P120 catenins is an important regulator of the cadherin-catenin adhesive complex that mediates cell-cell adhesion, controls transduction of signals that regulates cellular events including cell growth, differentiation, polarity, and cell migration (8).

Mutational and epigenetic inactivation of E-cad has been intensely implicated in the expansion and progression of cancer. In such a situation, p120 translocates to the cytosol where it exercises its oncogenic properties by abnormal regulation of Rho GTPases, depression of Kaiso (known also as ZBTB33) target genes, and growth factor receptor signaling (12). The first indications that p120 has oncogenic activity came from research in which p120 in the salivary along with mammary glands was conditionally deleted. Since then, p120 has been implicated in a number of cancers such as the tumors of the gastrointestinal tract, lung, pancreas, and so on; in which p120 every so often displays aberrant localization and reduction of expression (13).

Studies of p120 in OSCC have correlated its reduced expression with prognosis, depth of invasion, and poor survival. However, there are very few studies regarding its expression in epithelial dysplasia of the oral cavity, while no studies exist on apparently normal mucosa adjacent to OSCC (ANMAOSCC). Hence, this study aimed to evaluate and correlate the expression of p120 antibody in ANMAOSCC and OSCC.

## Material and Methods

This retrospective study included 300 paraffin-embedded tissue blocks comprising of histologically diagnosed cases of OSCC (n=150) and apparently normal mucosa adjacent to OSCC (ANMAOSCC, n=150). The specimens were taken from the surgical margins of excised lesional tissues that were retrieved from the archives of the Department of Oral Pathology and microbiology of Jaipur Dental College. Ethical approval was obtained from Institutional Ethics Committee of Jaipur Dental College, Jaipur (IEC/JDC/2023/M.No.(8)/Acad-82).

Inclusion Criteria:

Tissue blocks included in the study must have a confirmed histological diagnosis of Oral Squamous Cell Carcinoma (OSCC). Tissue blocks were sourced from surgical margins of excised lesional tissues.

Exclusion Criteria:

Tissue blocks lacking a histological diagnosis of OSCC, tissue blocks with insufficient or inadequate specimens for proper evaluation and cases where the histological diagnosis did not align with either OSCC or ANMAOSCC were excluded. Tissue blocks without accompanying clinical information relevant to the study, tissue blocks sourced from locations other than the surgical margins of excised lesional tissues, as specified in the inclusion criteria, were excluded. Cases with missing or incomplete data necessary for analysis were excluded.

Two tissue sections of 4µm thickness from the selected blocks were prepared on a soft tissue microtome (Leica RM2145) and taken onto amino propyl triethoxysilane (APES) coated slides. One section was stained with Hematoxylin and eosin. While the other slide was stained immunohistochemically (IHC) using anti –p120 catenin antibody (clone No. EP66; Catalog No. PR062; PathnSitu, Wayne, PA, USA).

IHC staining protocol: Slides were deparaffinized by heating on a slide warmer at 60ºC for 1 hour and treated with two changes of xylene for 10 minutes. The slides were treated with one change each of 100% alcohol followed by graded alcohol 90%, 80%, 70%, and 50% for 10 min each for dehydration. Slides were then rinsed with distilled water. A staining trough filled with Tris- EDTA (pH 9) along with the slides was placed in an EZ retriever system for three cycles at 96ºC for 6 minutes. After the cycles were complete the staining trough with the slides was gently taken out and cooled to room temperature. The slides were then washed in distilled water for 5 min followed by a TBS rinse for 5 min.

IHC staining procedure: Endogenous peroxidase activity was blocked by incubating with peroxidase block using PolyExel H2O2 for 10 minutes. Slides were washed with two changes of wash buffer (TBS) for 5 minutes each. Then the slides were incubated with anti –p120 catenin antibody for 45minutes in a humidified chamber. After that slides were washed with two changes of wash buffer (TBS) for 5 minutes each. PolyExel Target Binder was added to promote/enhance Ag-Ab reaction and incubated for 20 minutes in humidifying chamber. This was followed by TBS –two rinses for 5 minutes. Slides were incubated with Poly HRP for 30 minutes and it was followed by two changes in TBS for 5 min.

Incubation with freshly prepared substrate/chromogen solution of 3,3’ Diaminobenzidine (DAB) in provided buffer (by mixing 25µl concentrated DAB in 500µl of substrate buffer for 10 slides) was done for 10 minutes. This step enables visualization of antigen-antibody reaction as a brown-colored end product. After that slides were dipped in distilled water to stop the reaction.

The criterion for evaluating the immunoexpression, encompassing intensity, location, and percentage, was established based on a modified approach drawing from the techniques proposed by Lo Muzio *et al*. and Zhang *et al*. (Table 1). These modifications could enhance the accuracy or relevance of the immunoexpression evaluation for p120 catenin in the context of OSCC and ANMAOSCC.

**Table 1:**
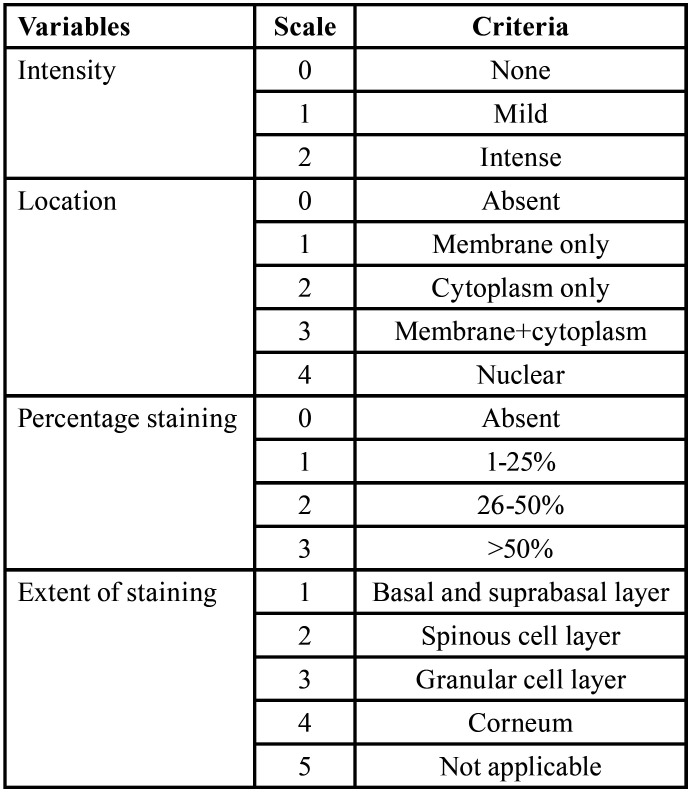
Criterion for evaluating the immunoexpression.

1. Intensity Evaluation: The modification may involve enhancements to the original method to better suit the specific nuances of the current study.

2. Location Evaluation: The modification entails refinements to the original method to capture additional details or variations in the location of p120 catenin expression. For example, it involved a more nuanced categorization of staining patterns, such as predominant membrane localization or combined membrane and cytoplasmic expression.

3. Percentage Evaluation: The modification may introduce adjustments to the percentage evaluation method, potentially refining the criteria for categorizing different levels of expression. This could involve a more granular scale, allowing for a more precise characterization of the extent of p120 catenin expression in the tissue samples.

4. Extent of Staining: The extent of staining, as a component of immunoexpression evaluation, involves assessing the spatial distribution of p120 catenin expression within the tissue samples based on different layers.

-Statistical analysis

The collected data was analyzed using the Statistical Package Social Sciences (SPSS) version 21. The statistical tool employed for these analyses was the Chi-square test, a non-parametric test commonly used to determine if there is a significant association between categorical variables. In this context, the Chi-square test was applied to investigate associations or differences in the intensity, location, percentage, and extent of p120 staining between OSCC and ANMAOSCC. Statistical significance was set at *p*≤0.05.

## Results

Table 2 revealed that the majority of the cases in the study were male, comprising nearly three-quarters (72.7%) of the total cases. Female cases constituted the remaining approximately one-quarter (27.3%) of the total. The distribution of diagnoses was evenly split between OSCC and ANMAOSCC, with each category representing half (50%) of the total cases.

**Table 2:**
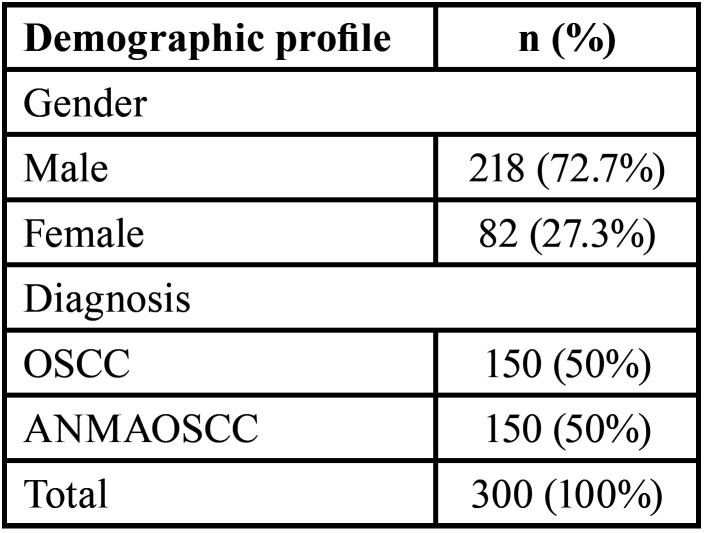
Demographic Distribution of the included cases.

**Figure 1:**
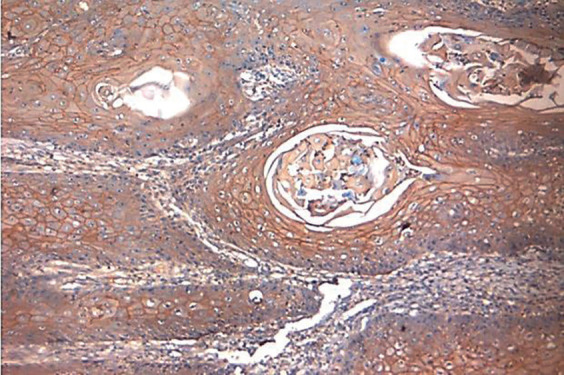
Photomicrograph of OSCC group showing areas of intense staining. 10X magnification.

**Figure 2:**
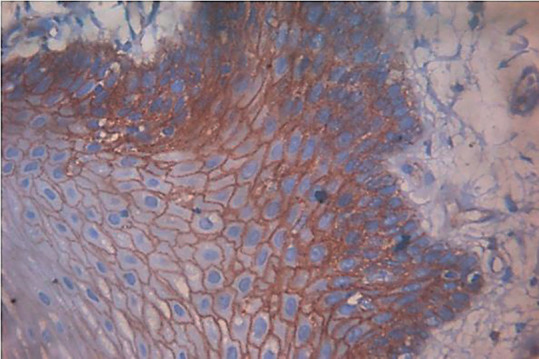
Photomicrograph of ANMAOSCC group showing intense membranous staining. 10X magnification.

Table 3 represents the comparison among groups with respect to the intensity of p120 staining. It was observed that significantly more than half of the OSCC specimens included in the study showed mild staining (58%), while 25% illustrated intense staining (Fig. 1). On the other hand, the ANMAOSCC specimens revealed intense staining in more than 80% (Fig. 2), with only 11.3% cases had mild staining.

**Table 3:**
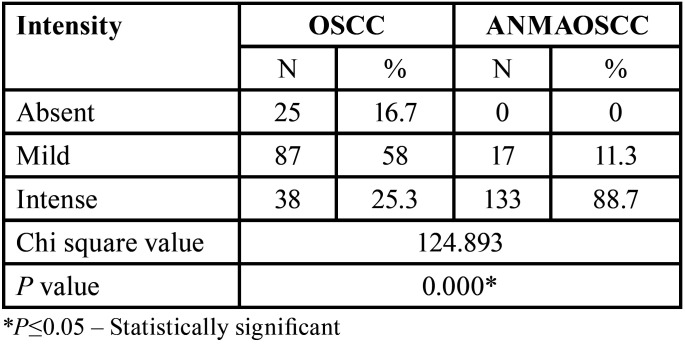
Distribution and comparison with respect to the intensity of p120 staining.

**Figure 3:**
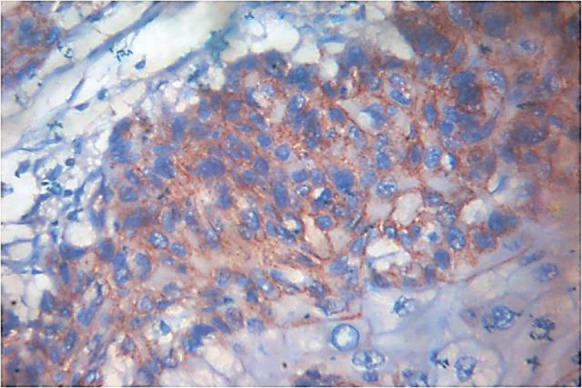
Photomicrograph of OSCC group showing areas of membranous and cytoplasmic staining. 40X magnification.

**Figure 4:**
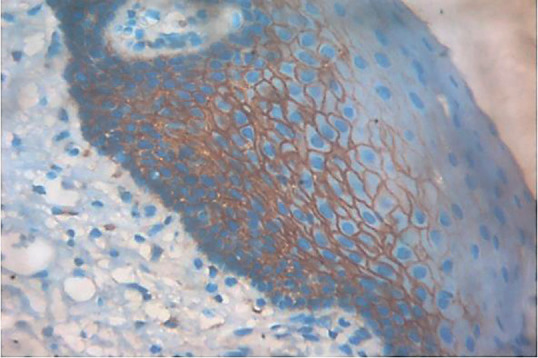
Photomicrograph of ANMAOSCCC membranous staining. 10X magnification.

Table 4 corresponds to the location of p120 staining and its comparison among groups. It was noted that significantly, the OSCC cases revealed membranous and cytoplasmic staining predominantly (76%) (Fig. 3) and also few cases didn’t showing staining (24%). However, in the ANMAOSCC cases, more than 90% of them demonstrated staining within the membrane only (91.3%) (Fig. 4) and a very small percentage of cases revealed membranous and cytoplasmic staining (8.7%).

**Table 4:**
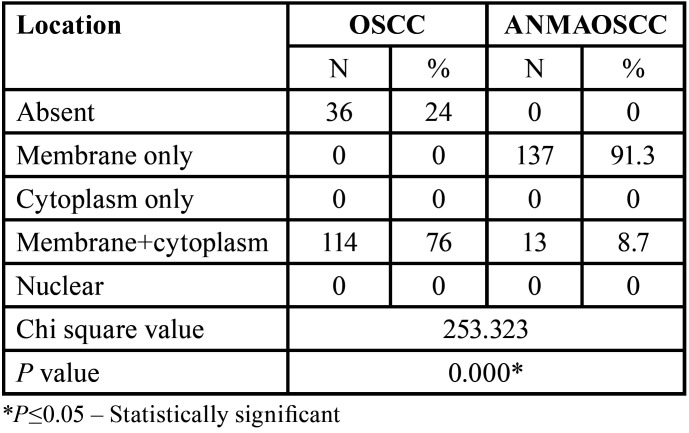
Distribution and comparison with respect to the location of p120 staining.

Comparison among groups with respect to the percentage of cells positive for p120 staining, it was observed that a comparable percentage of OSCC cases showed positive staining involving <25% of cells (32.7%) and 25-50% of cells (30.7%). Further, approximately 20 % of cases revealed positive staining in more than 50% of cells and small percentage of cases showed no staining (17.3%). When the ANMAOSCC cases were considered, it was observed that more than half of the cases preserved expression of staining by more than 50% of cells (Table 5). Further, the above comparisons were statistically significant.

**Table 5:**
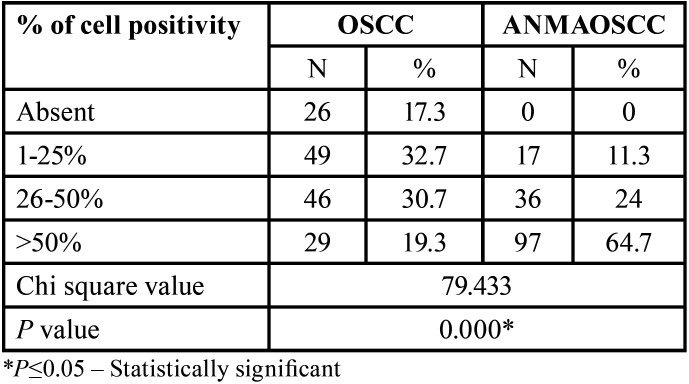
Distribution and comparison of cell percentage positive for p120 staining.

**Figure 5:**
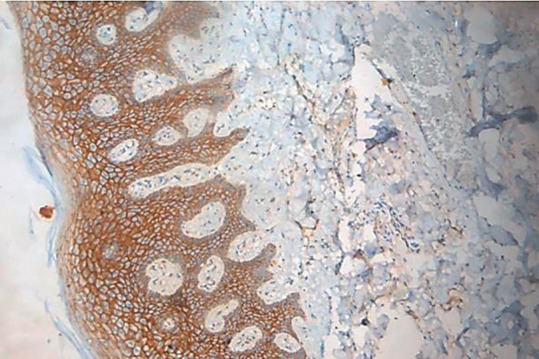
Photomicrograph of ANMAOSCC showing staining till granular layer. 10X magnification.

Comparison in regards to the extent of staining revealed a significant difference (*p*=0.000) among the ANMAOSCC cases. However, this criterion is not applicable to carcinoma cases due to the loss of function and architecture. A higher percentage of cases revealed staining up to granular cell layer (45.3%, Fig. 5) and 19.3 % of cases up to corneal cell layer. Further a considerable percentage of cases revealed staining only within the basal and suprabasal layers (24.7%). Moreover, a small percentage of cases showed spinous cell layer involvement (16%), (Table 6).

**Table 6:**
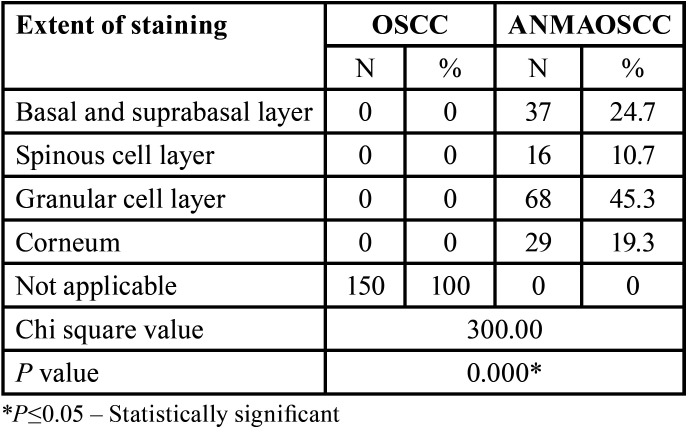
Distribution and comparison based on extent of p120 staining.

## Discussion

The classical role of p120 catenin as a structural protein stabilizing cadherin at the cell membrane has evolved, revealing its potential as a tumor suppressor (14-16). Loss or downregulation of p120 is evident in 50-60% of various cancers, impacting patient outcomes. Mouse model studies further support the tumor-suppressive role of p120, demonstrating its conditional deletion promoting tumor formation (17-19). Notably, OSCC often exhibits mislocalization of p120 from the membrane to cytoplasm, correlating with increased invasiveness, progression, and poor prognosis (20).

Modulation of cadherin by p120 is through direct action by binding to Juxtamembrane Domain (JMD) of the cytoplasmic tail or indirectly by regulation of Rho-GTP-ase signaling at adherins junction. Any mutations of JMD or altered Rho GTP-ase signaling increase the cadherin endocytosis predisposing to invasion and carcinogenesis. Also, p120 catenin loss has been associated with inflammation-driven carcinogenesis where it can activate NFK-β, which stimulates the release of growth factors and colony-stimulating factors that can recruit immature myeloid cells, thus generating a pro-invasive niche that interact and activate the carcinoma-associated fibroblasts leading to invasion. Various mechanisms have been implicated in the loss or downregulation of p120 catenin including gene mutation of CTNND1, transcriptional misregulation, epigenetic alteration, miRNA induced silencing, or degradation defects related to GSK 3β, casein kinease - 1α etc (21,22) or through Wnt signaling (23). In addition, Wu *et al*. (24) observed a positive correlation between the phosphorylation of p120 catenin in primary tumors with lymph node invasion in human breast cancer specimens.

Improvement of patient survival and to achieve the potential of molecular management of neoplasia mandates a better understanding of the biological nature of the disease which could facilitate the development of novel and more efficient treatment modalities for this disease (25). Biomarkers disclose the genetic and molecular variations related to early, intermediary, and late stages in the progression of oral carcinogenesis. These markers will refine our ability in predicting the biological course of oral cancer and thereby aid in the identification of individuals at high and or low risk of oral cancer development. There exist two studies on OSCC in literature and one in potentially malignant oral lesions (8,20). The study of this marker in apparently normal mucosa adjacent to OSCC along with molecular alterations has not been attempted so far. Therefore the present study aimed to evaluate the p120 expression in OSCC and apparently normal mucosa adjacent to OSCC.

The variation in the intensity of p120 in all the studies was statistically different (*p* <0.000) with intense stain seen in apparently normal mucosa adjacent to OSCC (88.7%). The OSCC group showed mild staining in around half of the cases (58%) with few showing intense staining (25.3%) and absence of staining (16.7%). Similarly, Lo Muzio *et al*. (20) demonstrated expression of p120 in 60-65% of OSCC cell lines and complete loss of expression in 15% of cases. In addition, Jiang *et al*. (26) observed very low expression of p120 (18±8%) in patients with locally advanced OSCC and in carcinomas with lymph node metastasis in comparison to early stage OSCC.

With respect to the location of p120, in the present study, there was an evident transition of p120 from membranous staining in the apparently normal mucosa adjacent to OSCC to membranous and cytoplasmic staining in OSCC cell lines. Around 76% of OSCC cases significantly showed membranous and cytoplasmic staining, 24% showed absence of staining and none of the cases showed nuclear staining. ANMAOSCC demonstrated mostly membranous staining in 91.3 % of cases. This cytoplasmic translocation of p120 in OSCC may be due to loss of E cadherin or due to phosphorylation at particular sites or any other post-translational modifications that lead to accumulation of p120 catenin in the cytoplasm. Changes in the p120 catenin isoforms may also be one of the reasons for the change in location and especially p120 catenin isoform-1 has been implicated in epithelial-mesenchymal transition and is seen in the cytoplasm.

In a study done by Ma *et al*. (8), it was reported that there was no expression of p120 in the epithelium of normal oral mucosal tissues. While in potentially malignant lesions, 51.5% of cases expressed p120 in the membrane, and a high cytoplasm expression is seen in 76.7% of OSCC cases. In addition, there was a propensity of lesions with a high membranous expression of p120 to develop into OSCC with odds of 3.43. Hence it was suggested that p120 affects cadherin adhesion by causing disruption of the adherens junction present in the epithelial cells and association of mislocalization of p120 with tumor progression in oral carcinoma.

In line with the present study findings, Sasaya *et al*. (27) also observed localization of p120 within the cell membrane of normal epithelial cells from basal to suprabasal layers and in the oral carcinoma tissue, the percentage of membrane positive cells were decreased and cytoplasm positive cells increased significantly. Further, nuclear staining was not detected in any of the cases. Since WNT signaling is indispensable for the development and maintenance of epithelial stem cells, the cytoplasmic localization and weak expression of p120-catenin at the basal cells membrane strengthen the understanding that catenins play a distinct role in oral epithelium physiology. However, Phattarataratip *et al*. (28) observed localization of p120 in the membrane of neoplastic cells in most of the salivary gland neoplasm.

Further, Jiang *et al*. (26) observed the lowest levels of p120 in the membrane and high levels in the nucleus of poorly differentiated neoplastic cells of OSCC. In contrast, well-differentiated OSCC had high levels of p120 in the membrane. In the noncancerous tissues, p120 was mainly localized in the plasma membrane. This high positivity of nuclear staining was also observed in locally advanced stages of OSCC and carcinomas with lymph node metastasis. Muzio *et al*. (20) also observed high membranous expression of P120 in all the normal oral mucosal specimens and cytoplasmic and nuclear staining is seen in 96% of OSCC cases. In addition, Li *et al*. (3) also observed reduced expression of p120 in the plasma membrane of OSCC compared with that in adjacent non-cancerous tongue epithelium.

No such study exists for ANMAOSCC or its comparison with OSCC, and only one study is present in the literature showing a similar trend which is seen in Esophageal squamous cell carcinoma (ESCC) and its adjacent normal esophageal epithelial mucosa, it was noted that the membrane expression of p120ctn in the ESCC samples was found to be significantly lesser than in the adjacent normal esophageal epithelial tissues (*P* = 0.041), while overall cellular expression was not different between the two tissue types (*P* = 0.787) (29). This was suggestive that the membrane localization of p120ctn may be beneficial to distinguish ESCC and normal esophageal tissue better than overall cellular expression.

In OSCC, five oral cell lines were used: NCTC 2544 (normal and immortalized keratinocytes), KB (dedifferentiated SCC cell line), OSC 20 (well-differentiated oral SCC cell line), CAL 33 (moderately differentiated oral SCC cell line), and CAL 27 (moderately differentiated oral SCC cell line) and evaluated for p120 expression by Muzio *et al*. (30). Thirty-five to forty percent of neoplastic cells (Cal 27, CAL 33, OSC 20, and KB) showed no positivity. There was an inverse correlation noted with the degree of differentiation. Immunohistochemically, it was found that 15% of OSCC showed loss of p120 catenin which was similar to that of our study (17.3%).

In ANMAOSCC, a preserved expression was seen with 65% of cells showing >50% expression while 35% showed reduced expression. No study was done with regard to apparently normal mucosa adjacent to OSCC. A study done on ESCC and apparently normal epithelial mucosa adjacent to esophageal carcinoma, reduced expressions of p120ctn ESCC tissues were evident as compared with adjacent normal esophageal epithelial tissues (29). In the present, the reduced expression evident in 35% of cases is of interest as it suggests early changes in cell adhesion and may serve as a field marker to predict recurrence and risk of developing second primaries.

In ANMAOSCC the staining was mainly seen till granular layer (45%) and corneal layer (19%) and this criterion was not applicable to the OSCC cases due to an already established carcinoma and lack of epithelial architecture. There was a progressive increase in the intensity of staining for p120 from basal and parabasal layers towards the intermediate spinous layer with less staining observed in the superficial layers for p120. Sasaya *et al*. (27) also observed positive staining for both p120 and beta-catenins from basal to suprabasal layers of normal oral epithelial cells. The present study acknowledges certain limitations. The study’s retrospective nature and reliance on paraffin-embedded tissue blocks might introduce selection bias. Limited to cases from a specific dental college’s archives, potentially impacting the generalizability of findings. Heterogeneity in histological features among OSCC cases may influence p120 expression, impacting the interpretation of results. The study concentrates solely on p120 catenin, neglecting potential interactions with other biomarkers that could provide a more comprehensive understanding of OSCC pathogenesis.

However, well-defined inclusion and exclusion criteria enhance the internal validity of the study by ensuring that tissue blocks with confirmed histological diagnoses of OSCC and suitable ANMAOSCC samples are included. A detailed and standardized immune-histochemical staining protocol is outlined, contributing to the reproducibility of results and facilitating potential comparisons with other studies using similar methodologies. While acknowledging these strengths, it’s essential to interpret the study findings within the context of its limitations to ensure a balanced understanding of the research’s scope and implications.

## Conclusions

The study revealed that p120 expression was prevalent in over 80% of oral squamous cell carcinoma (OSCC) cases, with 16.7% exhibiting no expression. In contrast, all cases of apparently normal mucosa adjacent to OSCC (ANMAOSCC) showed p120 expression. Notably, mislocalization of p120 occurred in over 70% of OSCC cases, shifting from membranous to cytoplasmic expression. OSCC displayed lower positivity for p120 (>50% of cells) compared to ANMAOSCC (64.7%). The staining pattern in ANMAOSCC extended to the granular layer (45%) and corneal layer (19%).

This study represents the first analysis of p120 expression in apparently normal mucosa adjacent to oral squamous cell carcinoma (OSCC), revealing robust membranous staining and heightened expression levels. The absence of staining in differentiated cells suggests a potential role of p120 in cellular differentiation. Consequently, p120 emerges as a crucial protein influencing the progression of metastasis, in line with the established understanding of adhesion molecule down-regulation as a key factor in initiating metastasis.
